# Outbreak investigation of an unknown gastrointestinal illness in District Victoria, Country Mala, 2016

**DOI:** 10.11604/pamj.supp.2021.40.2.30992

**Published:** 2021-12-15

**Authors:** Muhammad Asif Syed, Nigus Abebe Shumuye, Maria Anyorikeya, Nurbolot Usenbaev, Eva Mertens, Hedia Bellali, Elizeus Rutebemberwa

**Affiliations:** 1 Field Epidemiology and Laboratory Training Program (FELTP), Islamabad, Pakistan,; 2 Mekelle University, College of Veterinary Sciences, P.O. Box: 2084, Tigray, Ethiopia,; 3 Graduate School of Chinese Academic of Agricultural Sciences, Lanzhou Veterinary Research Institute, Gansu Province, China,; 4 Navrongo Health Research Centre, Navrongo, Ghana,; 5 Republican Center of Quarantine and Extremely dangerous diseases of Ministry of Health, Bishkek, Kyrgyzstan,; 6 Bernhard Nocht Institute for Tropical Medicine, Department of Infectious Disease Epidemiology, Bernhard-Nocht-Strasse 74, 20359 Hamburg, Germany,; 7 Global Partnership Initiated Biosecurity Academia for Controlling Health Threats (GIBACHT), Hamburg, Germany,; 8 Habib Thameur Hospital, Department of Clinical Epidemiology, Tunis, Tunisia,; 9 Medical Faculty of Tunis, Tunis El Manar University, Tunis, Tunisia,; 10 Programs, African Field Epidemiology Training Network, Kampala, Uganda,; 11 Makerere University College of Health Sciences, Kampala, Uganda,; 12 Global Partnership Initiated Biosecurity Academia for Controlling Health Threats (GIBACHT), Kampala, Uganda

**Keywords:** Case study, biosafety, biosecurity, outbreak investigation, epidemiology, risk communication, surveillance

## Abstract

This case study is based on an outbreak investigation conducted by multisectoral team from animal and public health offices in Kaktong (a remote village in Zhemgang District Bhutan) during July-September 2010. This outbreak caused by ingestion of infected cow meat which had died after a brief illness (bleeding of unclotted blood from nostrils). The owner of the affected cow had opened the carcass and dressed the meat, which he shared or sold within the village for human consumption. It simulates an epidemiological investigation including active and passive case finding, descriptive and analytical epidemiology, laboratory confirmation, risk communication with implementation of control measures. This case study is designed for the training of front-line public health professional, basic, intermediate and advanced level field epidemiology trainees. The case study will build the capacity of the trainees regarding investigating illnesses caused by animal-human interface.

## How to use this case study

### General instructions

This case study is an added resource for students of epidemiology and public health with specialised knowledge who need further information on the outbreak investigation with management and risk communication. The case study is ideally conducted in groups of about 10-20 participants under supervision of facilitator. Each student will participate in the case study by reading the paragraph on his/her turns. The facilitator will be responsible for engaging students in discussion, clarifying any confusing concepts or data analysis, and encouraging participants to think about the answers of the given questions. Notes for facilitator are coupled with each question in the facilitator version of case study with objective to aid facilitation.

### Target audience

This case study was designed for public health professional (medical doctors, nurses, environmental health officers or laboratory scientists etc.) and trainees of field epidemiology (frontline, intermediate and advance level)

### Prerequisites

Before using this case study, participants should have background knowledge on Anthrax (natural history of disease), public health surveillance and outbreak investigation.

### Time required

Approximately 3.5 hours

### Language

English

## Case study material

- Download the case study student guide (PDF - 550 KB)
- Request the case study facilitator guide: contact info@gibacht.org
